# How household chaos affects social withdrawal of rural children: the indirect role of executive function and receptive language ability

**DOI:** 10.3389/fpsyg.2023.1212426

**Published:** 2023-07-04

**Authors:** Shuwei Zhan, Jinna Guo

**Affiliations:** ^1^School of Education, Central China Normal University, Wuhan, Hubei Province, China; ^2^Shantou Teacher Development Center, Shantou, Guangdong Province, China

**Keywords:** household chaos, executive function, receptive language ability, social withdrawal, young Chinese children

## Abstract

Executive function (EF) and receptive language ability play an important role in the relationship between household chaos and social withdrawal. Young children are neglected in household chaos research and suffer from the negative outcomes of households in China. However, few studies have focused on the relationship between household chaos and social withdrawal in young Chinese children and the chain mediating effect of EF and receptive language ability. This study included 922 preschool-age children (62.58 ± 8.03 months) and their primary caregivers and their teachers were recruited from 21 rural preschools in Guangdong Province in China. Our results show a positive direct effect of household chaos and social withdrawal. Furthermore, an indirect sequential effect of household chaos and social withdrawal on EF and receptive language ability was found. Our findings (a) highlight the significance of paying more attention to household chaos and revealing a better understanding of the effect of EF and receptive language ability on households at an early age and (b) indicate that interventions should be provided to improve the home environment when children are young.

## 1. Introduction

Social withdrawal refers to children’s behavior of playing alone in an unfamiliar or familiar social environment (Rubin and Chronis-Tuscano, 2021). Children in social withdrawal may be at risk of later maladjustment (Guedeney et al., 2014) and further accumulation of human capital for national development (Chen and Liu, 2021). Studies have shown that numerous risk factors can affect children’s social withdrawal as they grow up in low-income environments (Li and Wong, 2015; Stuart et al., 2022). Examining the impact of early adversities on preschool children’s mental health is a new and developing area of research. Studies in Western countries emphasize that the degree of family clutter has a particularly high relationship with children’s social–emotional ability (Chia et al., 2021). As Berry and her colleagues showed that household disorganization in early childhood was predictive of worse cognitive and social outcomes at about age five (Berry et al., 2016). Poor living conditions early in life increase the risk of psychological problems in young children (Flouri et al., 2017), as well as the likelihood of continued exposure throughout life (Doom et al., 2018). However, few studies have focused on the Early experience of family chaos in rural Chinese children. Therefore, we can make more precise and accurate judgments for assisting children and families in China if we have a greater understanding of which and how family contextual elements impact children’s social withdrawal. It is crucial to look at how early household chaos affects Chinese children’s social withdrawal in rural areas.

Few studies have examined the underlying mechanisms, despite studies showing a link between early familial chaos and social withdrawal in children. Executive function (EF) refers to the psychological process in which individuals consciously control their thoughts, emotions, and behaviors when facing the target (Miyake et al., 2000). Studies have suggested that the association between family environment and adverse outcomes may be mediated by EF through linguistic skills (Berry et al., 2016). Therefore, the purpose of this study is to explore the mediation effects of EF and receptive language ability on the relationship between household chaos and social withdrawal.

### 1.1. Household chaos and social withdrawal among young children

The family is the most important proximal environment for the growth of children, as well as the micro context system of children’s development, which lays a foundation for their future cognitive, emotional, and social development (Fletcher and Wolfe, 2016; Liu et al., 2020). Previous studies mostly analyzed the impact on children’s physical and mental development from psychosocial characteristics such as family income, parental rearing style, and the parent–child relationship (Vernon-Feagans et al., 2016; Koutra et al., 2022). In recent years, the physical or material characteristics that affect children’s development (such as noise, chaos, etc.) have gradually attracted the attention of researchers (Akram and Shamama-Tus-Sabah, 2020). Together with various environmental pressures, household chaos is an important aspect of the physical microenvironment. Household chaos can produce adverse development results by interfering with the interaction between people and the environment (Marsh et al., 2020). Children living in a high-complexity family environment will have potential risks of low social ability, poor cognitive ability, etc. (Liang et al., 2020). Unlike urban children, rural preschool children live in rural areas where material resources are relatively scarce. Few research, however, has examined the link between social withdrawal among young Eastern rural children and household chaos.

### 1.2. Household chaos, EF, and social withdrawal

EF may be crucial in the relationship between household chaos and social withdrawal. Research had shown EF is a set of goal-oriented high-level cognitive abilities that can help children stop a reaction and adopt more appropriate behavior (Casey et al., 2014). Consequently, EF is assumed to play a significant role in the link between household chaos and behavioral problems. Di Norcia et al. (2015) found that EF, as a set of complex, higher-order cognitive processes, can help children manage their behavior in social situations and affect their social adaptation. A longitudinal study conducted by Selcuk et al. (2018), for example, showed that EF is an important predictor of social withdrawal in high-risk populations. On the other hand, household chaos will affect the development of children’s cognitive abilities. Since preschool children have not fully developed the attention and regulation ability to distinguish irrelevant stimuli, they are easily distracted by irresistible and changing external stimuli at home, resulting in lower EF throughout preschool education (Lillard et al., 2015; Vernon-Feagans et al., 2016). In addition, the EF can also mediate the relationship between parental rearing style, socioeconomic status (SES), etc., and individual behavior problems (Deer et al., 2020). For example, Lawson and Farah (2017) concluded from a study of 336 children between 6 and 15 years old that EF could partially mediate the relationship between SES and math achievement. As most studies focus on the effect of EF in the associations between household chaos and behavior problems from primary childhood to teenagers, few studies have examined whether EF plays a mediating role in the relationship between household chaos and social withdrawal in early children.

### 1.3. Household chaos, receptive language ability, and social withdrawal

It’s possible that the link between household chaos and social withdrawal depends heavily on receptive language ability. Receptive language ability refers to the ability to understand and interpret the transmitted information through language input processes such as listening or reading (Lyons, 2013). Language is an essential tool for human social communication. Children’s understanding of lexical meaning and usage directly affects the quality of children’s language expression, impacting their normal social communication (Blume et al., 2021).

According to previous studies, the family environment greatly impacts the development of children’s individual language abilities (Hammer et al., 2010; Lohndorf et al., 2019). An adverse living environment can affect the development of children’s language and early literacy through a certain mechanism (Berry et al., 2016). Rafferty et al. revealed that home environment, and greater family resources when children were newborns all predicted improvements in language ability (Rafferty et al., 2011). Hohm et al. (2007) showed that, at 10 months, both expressive and receptive language development were strongly related to cognitive and educational outcomes at age 10.

A few studies have further investigated the possible indirect role of the receptive language ability between household chaos and social withdrawal. The lack of an effective communication environment caused by the chaos of the family environment may be the cause of social withdrawal in rural preschool children (Vernon-Feagans et al., 2012; Zhan et al., 2021). The lack of receptive language ability interferes with the development of language expression ability and exacerbates the formation of social withdrawal behavior (Rubin and Coplan, 2004). Therefore, more evidence is needed to understand the role of receptive language ability in the link between household chaos and social withdrawal in rural preschool children.

### 1.4. Household chaos, EF, receptive language ability, and social withdrawal

Children’s social withdrawal behavior determines whether individuals have communication motivation which affects the development of their social skills in social interaction (Brei et al., 2014; Blume et al., 2021). Previous studies revealed that household chaos will affect the development of individual thinking, language, and social communication ability (Vernon-Feagans et al., 2016; Chimed-Ochir et al., 2022). Influencing factors of family chaos and psychological mechanisms have always been a concern by researchers. Obvious research showed that the integration stage of the understanding process activates the information consistent with the context through the activation procession or inhibition procession and forms an orderly and coherent psychological representation (Henry et al., 2011). Language acquisition and understanding are closely related to the human psychological mechanism and cognitive neural basis (Schuit et al., 2011; Gooch et al., 2016). However, the potential interrelationship between household chaos, EF, receptive language ability, and social withdrawal has not yet been well documented in China. Understanding this route better would enable us to support the following targeted treatments and support for preschoolers in remote regions with some data.

### 1.5. Current study

Although many studies have explored how household chaos is associated with behavior problems and the role of EF and language ability in these relationships, no research has established a connection between household chaos and social withdrawal at ages three to seven in young rural Chinese children and the effect of EF and receptive language ability. To expand the current knowledge, this study aims to (1) reveal the relationship between early household chaos and social withdrawal at age three to seven, (2) examine whether EF and receptive language ability play a chain mediating role in the relationship between household chaos and social withdrawal.

## 2. Materials and methods

### 2.1. Participants

There were 995 children recruited from 21 rural preschools in this project. We excluded 31 caregivers who did not give feedback on the questionnaire information, 30 caregivers who missed more than 3 questions in a questionnaire, and 12 children who were sick and failed to finish the assessment. Finally, a total of 166 teachers (all female, *M* = 29.31 years, SD = 8.10), and 922 children (*M* = 62.58 months, SD = 8.03, range = 40–84 months; 414 girls and 508 boys) and their caregivers participated in this study. In Guangdong province, a southern province of China, teachers, and kids were recruited using a deliberate random selection strategy. First, the Guangdong province government identified the cities as having below-average economic levels, and those cities were chosen. From the communities with below-average economic levels, 21 preschools were arbitrarily chosen, according to the school list given by the Board of Education. Second, 40–50 children were randomly selected in each preschool. Third, the families of the children in 21 rural preschools were looked into to ensure their parents’ yearly income was lower than the regional average. Children have been selected based on the following inclusion criteria: (a) the child is studying in preschool, (b) the child has a rural Hukou and has lived in the countryside, and (c) The child has no disabilities or intellectual disabilities. The participating educators were familiar with the children and worked with them for at least 4 months.

### 2.2. Procedure

The project director and graduate assistants (GAs) have explained the research aims, research process, and usage of the research tools to all the teachers and caregivers after school. To facilitate communication, all of the educators and caregivers signed consent papers and provided their contact information. In this study, the teachers and caregivers were asked to complete questionnaires. In addition, before distributing the questionnaire to the caregivers, the preschool teacher held a meeting to help the caregivers who did not understand the questionnaire’s content. The assessment of the child was carried out strictly as required in the preschools. The ethics approval has been obtained from the first author’s university.

Once agreed for their children to participate, caregivers completed a comprehensive questionnaire that included family demographic information and family environment. The teachers completed the children’s behavior questionnaire. After finishing the questionnaires, caregivers were instructed to send the packet to instructors, who then gave it to the GAs. To help with data collecting, six graduate assistants with educational psychology majors were trained. The GAs would try to establish a relationship with the youngster before the evaluation by conversing casually with them or engaging them in a brief game. The child’s receptive language ability and EF were assessed by the GAs. The child had the right to quit the study at any time. Most children were cooperative and completed the tasks successfully. Upon completing the whole assessment, the child was given a sticker as appreciation for his or her participation. In this study, we used Mandarin as the language of communication.

### 2.3. Measures

#### 2.3.1. Household chaos

Household chaos was assessed with the Chinese version of the Confusion, Hubbub, and Order Scale (CHAOS) (Matheny et al., 1995). The CHAOS was used to measure the degree of chaos, noise, and disorder in the family environment. The Chinese version of the CHAOS was culturally modified, standardized, and validated by Chang and her colleagues (Chang et al., 2016). The CHAOS was self-reported by parents and consisted of 15 items rated using a 4-point Likert scale ranging from 1 = nothing to 4 = a great deal. Sample items include, “We can usually find things when we need them” (reversed), “Our home is a good place to relax” (reversed), “You cannot hear yourself think in our home,” and “We always have many unnecessary arguments in our home.” The total score is high, indicating that the family environment is complex. Results of confirmatory factor analysis (CFA) supported the one-dimensional factor structure in this study of Chinese parents (*χ*^2^/*df* = 2.37, RMSEA = 0.04, CFI = 0.98, TLI = 0.97, SRMR = 0.03). The McDonald’s *ω* was 0.79 in this study.

#### 2.3.2. EF

The Chinese Head-Toes-Knees-Shoulders adapted from the HTKS were used for assessing children’s behavioral self-regulation, which required cognitive flexibility, working memory, and inhibitory control (McClelland and Cameron, 2012). During the first part of the task, children were instructed to follow paired behavioral commands (e.g., “touch your head”). Children were later instructed to do a behavior opposite of the verbal command (e.g., touching their toes when told to touch their heads). There are a total of 30 test items with scores of 0 (incorrect responses), 1 (self-correct responses), or 2 (correct responses) for each item. A self-correct is defined as the action of incorrect responses corrected by themselves. Total scores range from 0–60, with higher scores indicative of better EF and higher levels of behavioral self-regulation. The McDonald’s *ω* of HTKS in the present study was 0.87.

#### 2.3.3. Receptive language ability

The Chinese Peabody Picture Vocabulary Test-Revised (C-PPVT-R) (Lu and Liu, 2005), adapted based on the PPVT-R (Dunn and Dunn, 1981), was used for assessing Chinese preschool children’s receptive language ability. PPVT-R is a nationally normed measure that has been widely used in diverse samples of young children (Wong et al., 2008). The C-PPVT-R is intended for assessing the receptive language of Chinese children between the ages of 3 and 12 in Mandarin with a total of 125 items. Past research has shown that the C-PPVT-R demonstrates strong psychometric properties (e.g., a high degree of measurement reliability) (Hu et al., 2018). The McDonald’s ω of C-PPVT-R in the present study was 0.96. As a widely used measure of receptive language ability, C-PPVT-R is highly regarded for its objectivity and quick administration (Cheng et al., 2009).

#### 2.3.4. Social withdrawal

To evaluate children’s withdrawal behaviors, we used a questionnaire developed by Ye (2007). This questionnaire was reported by teachers and consisted of 12 items in three dimensions: shy silence (5 items, e.g., “He/She does not like to draw attention to himself”), active withdrawal (4 items, e.g., “He/She likes to play alone”) and passive withdrawal (3 items, e.g., “He/She will cry when friends do not play with him/her”). The responses are given on a 4-point Likert scale from 0 = never to 3 = always. The total score is the sum of the dimension scores. The higher the total score, the more withdrawn the child is. The scale has been widely used in China with good validity (Ye, 2007). Results of confirmatory factor analysis (CFA) supported the three-dimensional factor structure in this study of Chinese teachers (*χ*^2^/*df* = 3.20, RMSEA = 0.05, CFI = 0.93, TLI = 0.92, SRMR = 0.04). The McDonald’s *ω* was 0.85 in this study.

#### 2.3.5. Family SES

SES was measured by primary caregivers’ self-reported total household income. These indicators were collected in the caregivers’ survey. Annual income was divided into nine categories ranging from 1 = less than RMB 2000 (equivalent to $300) to 9 = RMB 100,000 or more (equivalent to $15,017 or more). Annual income is one of the most commonly used indicators of SES in developmental research (Cohen et al., 2006). We used caregivers’ annual family income as a reference to measure family SES because it is highly correlated with other SES indicators such as education and occupation (Li et al., 2019) and it can predict the rural family living condition directly. Research has shown that wealth may be a better measure of the financial resources available in that it is often a more accurate barometer of access to opportunities (Bradley and Corwyn, 2002). Considering that the ordinal education variable was treated as a continuous variable, the information loss is minimal, and the deviation is small. Therefore, SES was analyzed as a continuous variable in this study.

### 2.4. Statistical analysis

First, we used preliminary statistical analyses to analyze the differences in children’s social withdrawal behavior according to demographic variables. Second, descriptive statistical analysis and correlation analysis were carried out to describe the variables’ nature and relationships with one another. Third, we used the Mplus 8.3 to test the confirmatory factor analysis and serial mediating effect model.

## 3. Results

### 3.1. Descriptive information for characteristics

Table 1 shows the descriptive statistics (mean and standard deviation) of the main variables and the results of correlation analysis. Overall, EF and receptive language ability were negatively associated with children’s social withdrawal (*r* = −0.15, *p* < 0.001 and *r* = −0.16, *p* < 0.001, respectively), household chaos was positively associated with children’s social withdrawal (*r* = 0.09, *p* < 0.01) and negatively associated with EF and receptive language ability (*r* = −0.10, *p* < 0.01 and *r* = −0.09, *p* < 0.05, respectively). EF was positively associated with children’s receptive language ability (*r* = 0.53, *p* < 0.001). In addition, there is no significant gender difference in social withdrawal behavior (*t* = 0.88, *p* > 0.05).

**Table 1:** Descriptive statistics and correlations for study variables.

Variable	1	2	3	4	5	6	7
1. Gender	–						
2. Age	0.01	–					
3. SES	0.01	−0.07^*^	–				
4. Household chaos	0.01	0.08^*^	−0.08^*^	–			
5. EF	0.05	0.32^***^	0.13^***^	−0.10^**^	–		
6. Receptive language ability	−0.04	0.41^***^	0.10^**^	−0.09^*^	0.53^***^	–	
7. Social withdrawal	−0.03	−0.06	0.02	0.09^**^	−0.15^***^	−0.16^***^	–
*M*	–	62.58	5.82	1.93	22.29	32.34	20.69
SD	–	8.03	1.93	0.47	19.99	16.50	5.59

^a^Gender is coded as dichotomous variable (0 = boy, 1 = girl); ^*^p < 0.05, ^**^p < 0.01, ^***^p < 0.001.

### 3.2. Mediating role of EF and receptive language ability

**Figure 1:**
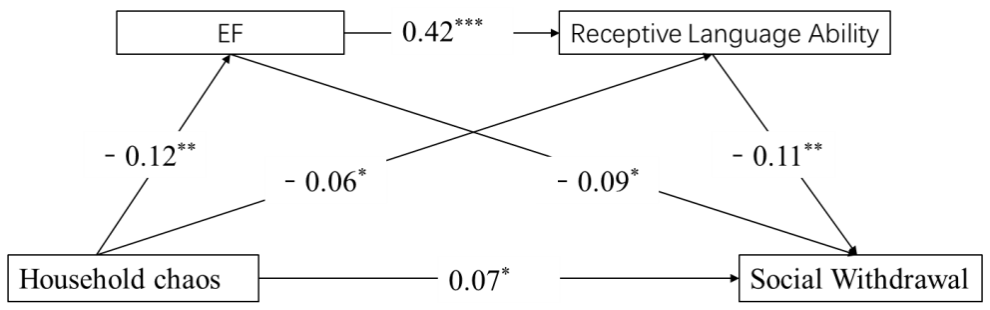
The chain mediation model of the association between household chaos, EF, receptive language ability, and social withdrawal (*N* = 922).The effects of demographic variables on the model are controlled for but not shown for simplicity.

We examined the hypothesis that household chaos might influence children’s social withdrawal through EF and receptive language ability. Figure 1 presents the pathway model. In this model, household chaos was intended to connect with EF, which would relate to receptive language ability and would affect children’s social withdrawal. As shown in Table 2, after controlling for children’s age and SES, household chaos was negatively related to EF (*β* = −0.12, *p* < 0.01) and receptive language ability (*β* = −0.06, *p* < 0.05), and these two factors were significantly associated with increased children’s social withdrawal (*β* = −0.09, *p* < 0.05; *β* = −0.11, *p* < 0.01).

**Table 2:** Sequential mediating models for children.

Dependent variable	Independent variable	*R-squared*	*β*	SE	*t*
Social withdrawal behavior		0.023			
	Age		−0.01	0.01	−2.06^*^
	SES		0.02	0.03	0.71
	Household chaos		0.10	0.03	2.91^**^
EF		0.136			
	Age		0.04	0.01	10.90^***^
	SES		0.14	0.03	4.57^***^
	Household chaos		−0.12	0.03	−3.76^**^
Receptive language ability		0.351			
	Age		0.04	0.01	10.08^***^
	SES		0.06	0.03	2.21^*^
	Household chaos		−0.06	0.03	−2.22^*^
	EF		0.42	0.03	14.76^***^
Social withdrawal behavior		0.038			
	Age		0.01	0.01	0.29
	SES		0.05	0.03	1.51
	Household chaos		0.07	0.03	2.22^*^
	EF		−0.09	0.04	−2.32^*^
	Receptive language ability		−0.11	0.04	−2.81^**^

All beta coefficients are standardized coefficients.

## 4. Discussion

The current study focused on rural preschool children’s social withdrawal and intended to investigate the relationship between household chaos and the social withdrawal of rural children, and further explore the potential relationship between household chaos, EF, receptive language ability, and social withdrawal. The finding is in accord with Bronfenbrenner’s theory and provided a possible explanation for how household chaos affects children’s social withdrawal. In sequence, EF and receptive language ability indirectly affect the pathways.

### 4.1. General condition of rural preschool children’s social withdrawal behavior

Our finding indicated that there is no significant gender difference between boys and girls in social withdrawal behavior, which is different from previous studies in Western societies (Bowker et al., 2020; Wood et al., 2021), which is consistent with recent studies of Chinese society (Ding et al., 2018). Research results in the West show that boys and girls have significant gender differences in social withdrawal, and girls’ social withdrawal is higher than boys’ (Janson and Mathiesen, 2008; Doey et al., 2014). The possible explanation is that there are gender role stereotypes in Western societies, where men are traditionally more dominant/assertive and women more passive/submissive (Doey et al., 2014). In China, however, social changes following 40 years of reform and opening up have changed the traditional preference for sons over daughters. Girls are no longer neglected by their families and can get the necessary emotional support and material help from their families. The gender gap that once existed in Chinese society is narrowing, and even families in relatively poor rural areas treat boys and girls on an equal basis (Zhou et al., 2016). Another possible reason is that the cultural environment will shape children’s social behavior while the same behavior may receive different evaluations and feedback in different cultural environments (Chen, 2010). Chinese culture emphasizes collectivism (Lu, 2019). There is no obvious gender difference in integrating into the social environment and actively interacting with others.

### 4.2. The relationship between household chaos and social withdrawal

Our findings revealed that household chaos could positively predict rural preschool children’s social withdrawal behavior. In other words, the higher the household chaos, the more serious the child’s social withdrawal problem. The results are in line with earlier research that suggested that early home learning environments were also negatively linked to their later behavior problems and social competence (Liang et al., 2020). Ferguson et al. (2013) came to a similar conclusion when they stated that human behavior and development are the results of interactions between people and their environment and that the physical environment significantly influences young children’s and adolescents’ cognitive and social–emotional development. As the proximal environment is directly experienced by children in their development, the family is the most important microsystem affecting children’s psychological development and the basis for children’s interaction with the external environment (Bronfenbrenner and Ceci, 1994). Qualitative information shows that parents are mostly engaged in physical labor and do not have enough energy to pay attention to raising their children in China’s rural areas, coupled with the phenomenon of multiple children in rural families, which aggravates the pressure of raising children and family chaos. Therefore, the chaos and disorder of the family environment will limit the opportunities for positive family communication and increase the possibility of individual social adaptation risks to a certain extent.

### 4.3. The mediating role of EF and receptive language ability

Our findings add insight into the mediating effect of EF on the relationship between household chaos and social withdrawal, as hypothesized, showing that the family material environment can affect an individual’s social communication ability by affecting the cognitive level. Rural preschool children’s perception of the material environment lacking in their family can affect an individual’s social communication ability by mobilizing the psychological process of EF. Previous studies also suggested that young children who lack material environment stimulation in rural families will lead to inability to solve problems in social situations (Jeon et al., 2014), which is difficult for them to concentrate and completely remember the rules in getting along with others and restraining their negative emotions, resulting in a series of social communication problems and social withdrawal behavior (Valadi et al., 2020). The executive function problems caused by the lack of material environment stimulation in rural families may lead to children’s inability to solve problems in social situations (Kuhn et al., 2021), which is difficult for them to concentrate and completely remember the rules in getting along with others and restraining their negative emotions, resulting in a series of social communication problems and social withdrawal behavior.

This study further shows that in addition to EF, receptive language ability also partially mediates the impact of household chaos on social withdrawal behavior. According to the family stress model, a complicated family environment (chaotic and crowded family life, etc.) will weaken the efficient interaction between parents and children. Many rural area families are nurtured only by grandparents because parents need to leave home to work in the city due to life pressure in China (Shi et al., 2021). Grandparents attach importance to parenting rather than education, making it difficult to give children a rich language stimulation environment (Fan and Guo, 2015), leading to low social communication ability and social withdrawal behavior. Speech restriction is very important for shyness and social withdrawal behavior. In a chaotic family environment, the lack of language expression caused by low receptive language ability weakens the motivation of social communication and intensifies social withdrawal behavior (Rubin and Coplan, 2004). Previous studies indicated that shy and withdrawn preschool children speak less to peers and adults than others in the classroom (Chow and Wehby, 2016), displaying poor performance across many developmental areas (Rubin and Chronis-Tuscano, 2021).

The key finding of this study extends past research by analyzing the potential chain mediating roles of EF and receptive language ability between household chaos and social withdrawal. Specifically, the influence of household chaos on children’s social withdrawal is through shaping the individual cognitive and language abilities. Previous studies have pointed out that EF can indirectly affect children’s adaptation and behavioral development by affecting language (Kaushanskaya et al., 2017). According to Information processing theory, individuals obtain information input from the environment (sensory information) and then use various cognitive processes (including executive functions) to operate, organize, and store this information. The development of language requires the use of EF, an indispensable cognitive processing mechanism, to absorb and integrate information (White et al., 2017). EF can activate relevant concepts, suppress or eliminate invalid information, think flexibly, process relevant working memory, and promote task execution. Preschool children are eager to interact with peers and adults. However, low EF level, low inhibitory control, cognitive transformation, and insufficient working memory will lead to insufficient language understanding, putting them in a passive position in social communication (Weiland et al., 2014). The specific psychological mechanism of household chaos affecting social withdrawal behavior is revealed, and the research on family environmental disadvantages and rural preschool children’s individual development and social adaptation is deepened.

### 4.4. Limitations and directions for future studies

Nonetheless, this study has some limitations. First, although the study is a cross-sectional design based on theory, it cannot accurately infer the causal relationship between variables. Therefore, future studies should use longitudinal designs to seek evidence for the causal assumptions made in this study. Second, the current study is limited to rural preschool children, their parents, and teachers in Guangdong Province. The findings cannot be extended to urban preschool children. Therefore, future studies are needed to explore such relationships in other parts of China and around the world. Thirdly, SES is the result of multiple factors. Our study only investigates the income of the caregivers and has not taken into account the education level, occupation, and other relevant contents of the parent. Therefore, it is necessary for future studies to comprehensively investigate more family information and enrich the first-hand data on the SES-related content of parents.

However, despite these limitations, this research indicates indirect effects of household chaos on children’s social withdrawal through the mediation of EF and receptive language ability. Social withdrawal is important for developing early childhood development programs. The mediating mechanisms of EF and receptive language ability may have implications for both theory and practice. These mediating mechanisms extend previous conceptual models regarding associations among family environment, EF, children’s language, and child performance in Chinese preschool contexts. By exploring these mechanisms, we develop a deeper understanding that EF and receptive language ability are major individual factors explaining how household chaos influences children’s social withdrawal. Future early childhood social skills studies should rely on the current study’s findings to consider how to positively affect children’s EF, receptive language ability, and living environment in the Chinese socio-cultural context, especially in rural China.

Therefore, we suggest that future studies can focus on proactive home-school partnerships, with lectures on parenting knowledge in preschool, to expose parents to the best practice in childrearing. A longitudinal study could better illustrate the relationship between household chaos, EF, receptive language ability, and young children’s emotional outcomes. In addition, the role played by the various components of executive function can be explored in the future. The difference between receptive and expressive words in young children’s social withdrawal is also an interesting topic for future research.

## 5. Conclusion

Our finding revealed a positive association between household chaos and social withdrawal among young rural children and the chain mediating pathway (i.e., via EF and receptive language ability). The findings show that early household chaos has to be given greater consideration and that early EF and receptive language abilities are important. Given the importance of EF and receptive language ability, prevention, and intervention programs need to be considered to reduce social withdrawal.

## Data availability statement

The original contributions presented in the study are included in the article/supplementary material, further inquiries can be directed to the corresponding author.

## Ethics statement

The studies involving human participants were reviewed and approved by Anhui Normal University. Written informed consent to participate in this study was provided by the participants’ legal guardian/next of kin.

## Author contributions

All authors listed have made a substantial, direct, and intellectual contribution to the work and approved it for publication.

## Funding

This research has been funded by the National Social Science Fund Educational Project (Number: BHA190149).

## Conflict of interest

The authors declare that the research was conducted in the absence of any commercial or financial relationships that could be construed as a potential conflict of interest.

## Publisher’s note

All claims expressed in this article are solely those of the authors and do not necessarily represent those of their affiliated organizations, or those of the publisher, the editors and the reviewers. Any product that may be evaluated in this article, or claim that may be made by its manufacturer, is not guaranteed or endorsed by the publisher.
